# Structural basis for acceptor RNA substrate selectivity of the 3′ terminal uridylyl transferase Tailor

**DOI:** 10.1093/nar/gky1164

**Published:** 2018-11-20

**Authors:** Alena Kroupova, Anastasia Ivaşcu, Madalena M Reimão-Pinto, Stefan L Ameres, Martin Jinek

**Affiliations:** 1 Department of Biochemistry, University of Zurich, Zurich 8057, Switzerland; 2 Institute of Molecular Biotechnology, IMBA, Vienna Biocenter Campus (VBC), Vienna 1030, Austria

## Abstract

Non-templated 3′-uridylation of RNAs has emerged as an important mechanism for regulating the processing, stability and biological function of eukaryotic transcripts. In *Drosophila*, oligouridine tailing by the terminal uridylyl transferase (TUTase) Tailor of numerous RNAs induces their degradation by the exonuclease Dis3L2, which serves functional roles in RNA surveillance and mirtron RNA biogenesis. Tailor preferentially uridylates RNAs terminating in guanosine or uridine nucleotides but the structural basis underpinning its RNA substrate selectivity is unknown. Here, we report crystal structures of Tailor bound to a donor substrate analog or mono- and oligouridylated RNA products. These structures reveal specific amino acid residues involved in donor and acceptor substrate recognition, and complementary biochemical assays confirm the critical role of an active site arginine in conferring selectivity toward 3′-guanosine terminated RNAs. Notably, conservation of these active site features suggests that other eukaryotic TUTases, including mammalian TUT4 and TUT7, might exhibit similar, hitherto unknown, substrate selectivity. Together, these studies provide critical insights into the specificity of 3′-uridylation in eukaryotic post-transcriptional gene regulation.

## INTRODUCTION

Post-transcriptional RNA modifications have emerged as important regulatory mechanisms that control the biogenesis, function and stability of RNA transcripts in eukaryotic cells (1). These ‘epitranscriptomic’ marks include base modifications such as N6-adenosine methylation (2,3), as well as the addition of non-templated nucleotides to the 3′ termini of RNAs (RNA tailing), which is one of the most frequent RNA modifications. 3′-Terminal polyadenylation of mRNAs confers stability and is required for efficient translation, while oligoadenylation of aberrant RNA transcripts stimulates their 3′–5′ exonucleolytic degradation by the exosome complex (4,5). In turn, recent studies have revealed that 3′-uridylation of both coding and non-coding RNAs is also widespread and serves important functions in their processing and decay (6–8).

RNA tailing is catalyzed by template-independent terminal ribonucleotidyl transferases belonging to the DNA polymerase beta superfamily (9), with most eukaryotic genomes encoding multiple enzymes with distinct specificities for the donor nucleotide triphosphate and acceptor RNAs (10,11). These comprise both canonical poly(A) polymerases (PAPs), responsible for generating poly(A) tails of mRNAs, as well as non-canonical PAPs (ncPAPs) which include terminal uridylyl transferases (TUTases) that catalyze the 3′-terminal addition of uridine residues to RNA (12). Some ncPAPs/TUTases exhibit mixed nucleotide donor specificities, catalyzing both 3′ adenylation and uridylation (13,14). In recent years, numerous studies have revealed that 3′-terminal RNA uridylation plays important functional roles (15). U6 small nuclear RNA (U6 snRNA) undergoes 3′ uridylation by TUT1 during its maturation, which is essential for its function in mRNA splicing (16). In fission yeast, the TUTase Cid1 catalyzes 3′ uridylation of a subset of canonical polyadenylated mRNAs, which subsequently undergo LSm1–7-dependent decapping and decay (17). Similarly, sequencing studies have detected low levels of 3′ uridylation in the majority of mRNA transcripts in mammalian cells (18), where uridylation by TUT4 or TUT7 enzymes (TUT4/7) contributes to global mRNA turnover by targeting mRNAs with short (<20 nt) polyA tails for degradation (19). 3′ uridylation also plays an essential role in promoting the degradation of replication-dependent histone mRNAs at the end of the S-phase (20,21).

The processing and stability of microRNAs (miRNAs) or their precursors is extensively regulated by 3′ uridylation in both animal and plant cells. Monouridylation of a subset of animal miRNA precursors, including group II pre-let-7 RNAs, by TUT4/7 restores the 3′ two-nucleotide overhang necessary for their downstream processing by the endonuclease Dicer (22). Conversely, processive oligouridylation of pre-let-7 RNAs by TUT4/7, facilitated by direct interaction with the RNA binding protein Lin28, inhibits let-7 miRNA maturation by promoting pre-let-7 degradation, thereby contributing to the maintenance of pluripotency in stem cells (23,24). 3′ oligouridylation is also implicated in the destabilization of mature miRNAs upon binding highly complementary targets in both *Drosophila* and mammalian cells. Oligouridylated RNAs become efficient substrates for degradation by the processive 3′-5′ exonuclease Dis3L2, a homolog of the catalytically active Dis3L subunit of the exosome complex (25–28). Dis3L2 specifically recognizes 3′-oligoU tails, which facilitates initiation of processive RNA hydrolysis and enables degradation of structured RNA substrates (29). Thus, an emerging consensus suggests that 3′ oligouridylation generally functions as a destabilizing mark that directs both coding and non-coding RNAs for rapid decay. In this way, this RNA modification not only contributes to global RNA turnover but also plays a role in the surveillance and quality control of the cellular transcriptome.

In *Drosophila*, the Dis3L2 ortholog (DmDis3L2) physically associates with the TUTase Tailor, forming the terminal RNA uridylation-mediated processing (TRUMP) complex (30,31). Tailor has been shown to catalyze 3′ uridylation of mirtrons, hairpin RNAs that arise by splicing of short introns followed by lariat debranching, thus inducing their degradation. Due to its intrinsic selectivity for RNAs containing 3′-terminal guanosine nucleotides, Tailor preferentially recognizes mirtron 3′ ends (terminated by the 3′-AG splicing acceptor sequence), while canonical pre-miRNAs, which are depleted in 3′ guanosines, avoid 3′ uridylation (32,33). In this way, the combined activities of the TRUMP complex shape the mature miRNA pool in *Drosophila* and may restrict *de novo* emergence of miRNAs. In addition to their function in miRNA biogenesis and surveillance, the activities of Tailor and DmDis3L2 within the TRUMP complex have also been implicated in the quality control of polymerase III transcripts (30), as was shown for mammalian Dis3L2 orthologs (34,35).

Although eukaryotic TUTases have been subject to extensive structural studies (14,36–42), the molecular basis for the 3′-terminal acceptor nucleotide specificity of Tailor is not known. Here we report crystal structures of *Drosophila melanogaster* Tailor (DmTailor) in complexes with a donor substrate mimic or mono- and oligouridylated RNA products, revealing the structural basis for donor UTP and acceptor RNA substrate recognition. Using biochemical experiments and structure-guided mutagenesis, we show that specific features of the DmTailor active site contribute to the 3′-G and 3′-U selectivity. Together, these results reveal a mechanistic basis for the preferential activity of Tailor toward mirtron RNA substrates and oligouridylated Pol III transcripts. As such, our studies provide a structural framework for understanding the functional roles of the TRUMP complex in RNA processing and surveillance, and have implications for the enzymatic activities of mammalian TUTases.

## MATERIALS AND METHODS

### DmTailor expression and purification

All experiments were performed with a truncated construct of *Drosophila melanogaster* Tailor (DmTailor) isoform B spanning residues 180–560 (DmTailor^180–560^). DNA encoding the construct was cloned into the UC Berkeley MacroLab 438-C vector (gift from Scott Gradia, Addgene plasmid #55220) using ligation-independent cloning. The resulting fusion protein containing an N-terminal His_6_ tag, a maltose binding protein (MBP), and a TEV-protease cleavage site was expressed in Sf9 insect cells using the Bac-to-Bac Baculovirus expression system (Invitrogen). Cells were harvested 60 h post infection and lysed by sonication in lysis buffer (20 mM HEPES, pH 8.0, 150 mM NaCl, 0.1% Tween, 2 mM MgCl_2_, 5 mM imidazole) supplemented with cOmplete™ Protease Inhibitor Cocktail (Roche). The lysate was clarified by centrifugation for 30 minutes at 30 000 g at 4°C and subsequently applied to a Ni-NTA Superflow resin (QIAGEN) and eluted with buffer containing 20 mM HEPES, pH 8.0, 500 mM NaCl, 2 mM MgCl_2_ and 250 mM imidazole. The fusion tag was removed by TEV protease during an overnight dialysis at 4°C against buffer containing 20 mM HEPES, pH 7.5, 150 mM NaCl and 2 mM MgCl_2_. The resulting solution was passed over a 5 ml Ni-NTA Superflow (QIAGEN) column to remove any uncleaved protein and the His_6_-MBP tag. The protein-containing fractions were concentrated (Amicon Ultra centrifugal filter, MWCO 10 kDa, Sigma) and further purified by size-exclusion chromatography on a Superdex 200 column (GE Healthcare) in buffer containing 20 mM HEPES, pH 7.0, 150 mM KCl, 2 mM MgCl_2_ and 1 mM DTT. A 5 ml amylose resin (New England Biolabs) cartridge was attached in line to remove residual MBP. Peak fractions containing DmTailor were concentrated (Amicon Ultra centrifugal filter, MWCO 10 kDa, Sigma) to 12 mg ml^−1^, flash frozen in liquid nitrogen and stored at –80°C. The point mutants of DmTailor^180–560^ were generated by QuickChange site-directed mutagenesis and verified by DNA sequencing. The mutants were expressed and purified as described above for the wild-type DmTailor^180–560^ with the exception of the D280A mutant for which the salt concentration was maintained at 250 mM in buffers for dialysis and all subsequent steps.

### Crystallization and structure determination

Crystals of DmTailor^180–560^ bound to uridine-5′-[(α,β)-imido]triphosphate (UMPNPP) were obtained using the hanging drop vapour diffusion method at 20°C. Purified DmTailor^180–560^ at a concentration of 10.8 mg ml^−1^ was pre-incubated with 1 mM UMPNPP (Jena Bioscience). Initial crystals were obtained by mixing equal volumes of protein-ligand solution with the reservoir solution containing 20% glycerol ethoxylate. The size and shape of the initial crystals was improved by micro-seeding. The crystals were transferred to a solution of 30% (v/v) glycerol ethoxylate (average *M*_*n*_ ∼1000, Molecular Dimensions) and 10% (v/v) ethylene glycol for cryoprotection before being flash-cooled in liquid nitrogen.

Crystals of DmTailor^180–560^ bound to the dinucleotide GpU were grown using the sitting drop vapour diffusion method at 20°C. DmTailor at a concentration of 5.6 mg ml^−1^ was pre-incubated with 1 mM GpU (IBA Lifesciences). Initial crystals were obtained by mixing equal volumes of protein–ligand solution with the reservoir solution containing 30% (v/v) glycerol ethoxylate and 60 mM sodium malonate, pH 7.0. The size and shape of the initial crystals was improved by micro-seeding. The crystals were transferred to a solution containing 40% (v/v) glycerol ethoxylate, 64 mM sodium malonate, pH 7.0, and 1 mM GpU for cryoprotection before being flash-cooled in liquid nitrogen.

Crystals of DmTailor^180–560^ bound to the hexanucleotide CACAGU were grown using the hanging drop vapour diffusion method at 20°C. Tailor at a concentration of 5 mg ml^−1^ was pre-incubated with 0.13 mM 5′-CACAGU-3′ RNA (Integrated DNA Technologies). The crystals were obtained by mixing equal volumes of protein–RNA solution with the reservoir solution containing 35% (v/v) glycerol ethoxylate and 75 mM sodium malonate, pH7.0. The crystals were transferred to a solution of 40% (v/v) glycerol ethoxylate, 75 mM sodium malonate, pH 7.0, and 0.13 mM CACAGU RNA for cryoprotection before being flash-cooled in liquid nitrogen.

Crystals of DmTailor^180–560^ bound to U_6_ RNA were grown using the sitting drop vapor diffusion method at 20°C. Tailor at a concentration of 5 mg ml^−1^ was pre-incubated with 0.26 mM U_6_ RNA (Integrated DNA Technologies). The crystals were obtained by mixing equal volumes of protein-RNA solution with the reservoir solution containing 35% (v/v) glycerol ethoxylate and 90 mM sodium malonate, pH 7.0. The crystals were transferred to a solution of 40% (v/v) glycerol ethoxylate, 90 mM sodium malonate, pH 7.0 and 0.26 mM U_6_ RNA for cryoprotection before being flash-cooled in liquid nitrogen.

X-ray diffraction data were collected at beamline X06DA (PXIII) of the Swiss Light Source (Paul Scherrer Institute, Villigen, Switzerland). The data were processed using XDS (43). All crystals belonged to space group *P*3_1_21, with one molecule in the asymmetric unit, and diffracted to resolutions of 1.9 Å (DmTailor^180–560^–UMPNPP complex), 2.0 Å (DmTailor^180–56^–GpU complex), 1.85 Å (DmTailor^180–560^–CACAGU complex) or 2.0 Å (DmTailor^180–560^–U_6_ complex). The structure of the DmTailor^180–560^–UMPNPP complex was solved by molecular replacement in Phenix Phaser (44) using the structure of the catalytic module of human TUT7 (PDB: 5W0B) as a search model. The remaining structures were solved by molecular replacement using the atomic coordinates of the DmTailor^180–560^–UMPNPP complex as search model. The initial building of the model was done using Phenix.Autobuild (45), finished manually in Coot (46) and refined in Phenix.Refine (47). The final DmTailor^180–560^-UMPNPP atomic model contains residues 202–417, 419–549, UMPNPP and one Mg^2+^ ion. The final DmTailor-GpU model contains residues 198–417, 419–548 and the dinucleotide GpU. The final DmTailor-CACAGU model contains residues 198–415, 420–548, four 3′-terminal nucleotides of the RNA substrate (CAGU) and two Mg^2+^ ions. The final DmTailor-U_6_ model contains residues 195–416, 419–548, four 3′-terminal nucleotides of the RNA substrate and two Mg^2+^ ions. Structural superpositions were performed using DALI pairwise alignment (48). The annealed omit maps were generated for the UMPNPP/GpU/CACAGU/U_6_ ligands using phenix.composite_omit_map (49). The electrostatic surface potential was generated using the Adaptive Poisson-Boltzmann Solver (APBS) plugin (50) in Pymol (Schrödinger, LLC).

### Multiple sequence alignment

The multiple sequence alignment was generated using MAFFT version 7 (51) and visualized using Jalview (52).

### *In vitro* TUTase activity assays

**Figure 1.**
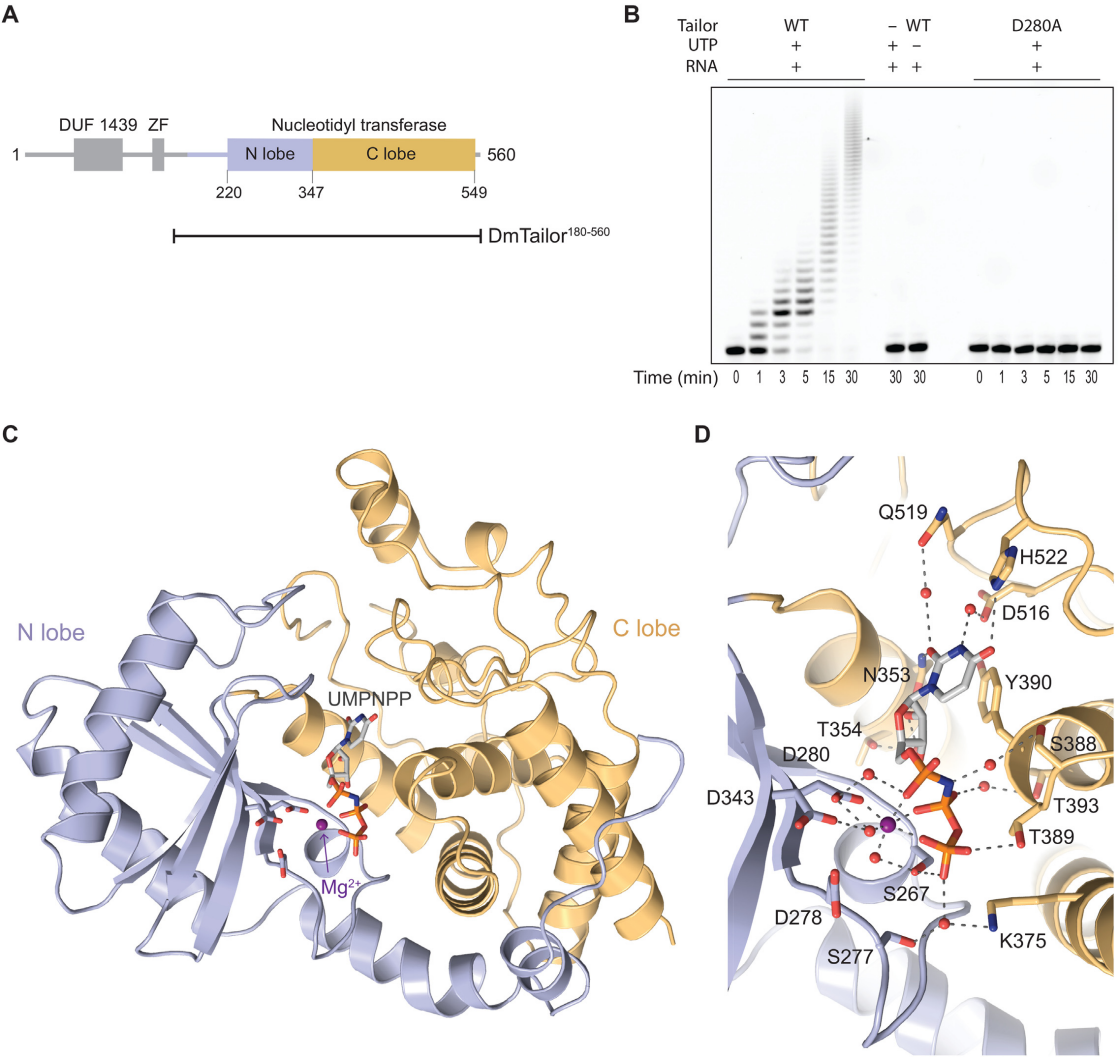
Structure of DmTailor in complex with the donor substrate mimic UMPNPP. (**A**) Schematic representation of the domain organization of DmTailor. DmTailor contains an N-terminal DUF1439 domain, a putative zinc finger (ZF) and a C-terminal nucleotidyl transferase domain consisting of the N-terminal lobe (N-lobe) and the C-terminal lobe (C-lobe). The N-terminally truncated construct DmTailor^180–560^ used throughout this work is indicated with a black line. (**B**) *In vitro* uridylation assay with wild-type (WT) DmTailor^180–560^ and catalytic mutant D280A. (**C**) Crystal structure of DmTailor^180–560^ bound to UMPNPP and Mg^2+^. The UMPNPP substrate analog, shown as grey sticks, and the Mg^2+^ ion, shown as a purple sphere, are bound in the active site formed between the N-lobe (blue) and the C-lobe (yellow). (**D**) Interactions within the active site. DmTailor is colored as in (C), with interacting residues shown in stick format. Hydrogen bonds and metal coordination bonds are shown as grey dashed lines, water molecules are shown as red spheres.

A 22-nt single stranded RNA oligonucleotide derived from the miR-1003 stem-loop (Supplementary Table S1, miR-1003–3p-G) and labeled with an Atto532 dye at its 5′ end was used as a substrate for the *in vitro* tailing assays. The reaction mixtures (final volume 40 μl) for the assay in Figure 1 contained 0.1 μM of WT or D280A DmTailor^180–560^, 1 μM RNA substrate and 0.5 mM UTP in a reaction buffer containing 20 mM HEPES, pH 7.0, 150 mM KCl, 2 mM MgCl_2_, 1 mM DTT, 0.05% Tween-20. A zero time point was collected before the addition of the enzyme into the reaction. The reactions were incubated at 27°C and 5 μl aliquots were collected at 1, 3, 5, 15 and 30 min. Reactions were stopped by the addition of EDTA to a final concentration of 90 mM and a 2× RNA loading dye (95% formamide, 5 mM EDTA, pH 8.0, 0.025% SDS). The samples were heated at 95°C for 5 min and resolved on a denaturing (7 M urea) 18% polyacrylamide gel. The Atto532 labeled RNA was visualized using a Typhoon FLA 9500 gel imager (GE Healthcare).

For the substrate-specificity assays in Figure 3, the tailing reactions were set up as above with minor modifications. Four RNA substrates were used differing only in the 3′-terminal nucleotide (Supplementary Table S1). The final concentration of RNA substrate was 1 μM. The final protein concentration was optimized depending on the activities of the mutants to allow for observation of the addition of the first nucleotide in the tailing assays. For WT, R327K and Q519A DmTailor^180–560^, the final concentration was 0.05 μM compared to 1 μM for the R327A mutant. The gels were quantified using the Image Lab Software (Bio-Rad) and the data analyzed and fitted with an exponential one-phase decay equation using the GraphPad Prism6 Software.

### Sequencing-based activity assay

#### Tailing assay

Assays were performed as described previously (32), with minor modifications. Briefly, tailing reactions were performed using physiological concentrations of all four rNTPs (0.5 mM UTP, 0.5 mM GTP, 0.3 mM CTP and 3 mM ATP), 10 nM 5′-^32^P-radiolabeled RNA substrate and 50 nM recombinant protein in a total volume of 10 μl. Reactions were carried out at 25°C and 2 μl aliquots were collected from the tailing reaction at 2, 5 and 10 min. For phosphor imaging, reactions were stopped in 8 μl formamide loading buffer (95% formamide, 18 mM EDTA, 0.025% SDS, Xylene Cyanol and Bromphenol Blue; Ambion). Samples were boiled at 95°C for 5 min and separated on a 15% denaturing polyacrylamide gel, dried, exposed to storage phosphor screen (PerkinElmer), and imaged using an Amersham Typhoon IP Biomolecular Imager (GE Healthcare Life Sciences). For generation of high-throughput sequencing libraries, reactions were stopped in 150 μl SDS-containing buffer (0.3 M NaCl, 0.1% SDS) and RNA was extracted with phenol–chloroform.

#### Library preparation

High-throughput sequencing libraries were performed as described previously (32), with minor modifications. Briefly, the RNA substrate was subjected to 3′ adapter ligation as described for small RNA cloning (53); the ligated substrate was extracted with phenol-chloroform and reverse-transcribed using SuperScript III Reverse Transcriptase (Invitrogen). The cDNA sample was PCR amplified using the KAPA Real-Time Library Amplification Kit (PeqLab) and size-selected in a 2% low-range ultra agarose gel (BioRad) as previously described (53). Libraries were subjected to quality control, multiplexed at equimolar ratios and SR50 sequenced on an HiSeq 2000 instrument (Illumina) performed by the CSF NextGen Sequencing facility (Vienna, Austria, www.vbcf.ac.at). Two technical replicates per time-point and per-condition were prepared and sequenced. See Supplementary Table S2 for sequencing statistics.

#### Data analysis

Analyses were performed as described previously (32). Briefly, after library demultiplexing, sequences were recovered by adapter clipping by cutting once with Cutadapt v1.2.1 (54). The random 4-mers on the 5′ linker were retained for the analysis and the 3′ linker sequence was removed with fastx_trimmer from the fastx-toolkit v0.0.13 (http://hannonlab.cshl.edu/fastx_toolkit/). Only reads that contained ≥4 nt remaining sequence and no ambiguous nucleotide were considered further; a minimum sequencing quality of 20 (PHRED) was required for all nucleotides. The nucleotides following the 5′ linker random 4-mers were considered as tailed reads. The total count of each substrate and respective tail length and nt identity was computed (for details see (32)). The average tailed reads of the two experimental replicates for each time point and each condition was considered for further analysis. Overall tail composition was determined by calculating the absolute number of untemplated nucleotides A, T, C and G at the 10 min time point, irrespective of substrate identity and tail length; reported values correspond to the average of two experimental replicates. Statistical test and plots were performed using GraphPad Prism v7.0d or Microsoft Excel v16.16.1.

## RESULTS AND DISCUSSION

### Structure and donor substrate recognition of DmTailor

DmTailor consists of a conserved nucleotidyl transferase domain located in its C-terminal part, while its N-terminal region contains a DUF1439 domain and a putative zinc finger domain (Figure 1A). The DUF1439 domain mediates direct interactions with the exonuclease DmDis3L2 but has no effect on the uridylyl transferase activity of DmTailor (30,31). Similarly, mutations in the putative zinc finger do not perturb the activity of DmTailor (31). To investigate the molecular mechanism underlying its substrate specificity, we set out to crystallize DmTailor in complexes with its substrates. Full-length DmTailor could not be expressed in recombinant form and purified (data not shown). Instead, we identified an N-terminally truncated construct spanning residues 180–560 (DmTailor^180–560^) that could be purified to homogeneity in sufficient quantity and quality for crystallization. The truncated construct encompasses the entire predicted nucleotidyl transferase domain (residues 220–549) and exhibits uridylation activity *in vitro*, which is abrogated by introducing a point mutation (D280A) in a conserved aspartate predicted to coordinate a catalytic divalent cation (Figure 1A and B). The catalytically active wild-type (WT) DmTailor^180–560^ construct was used throughout this work for crystallization as well as biochemical characterization of substrate recognition.

We initially crystallized DmTailor^180–560^ in complex with the non-reactive UTP donor substrate analog UMPNPP, in the presence of Mg^2+^ ions (DmTailor^180–560^-UMPNPP). The structure was solved by molecular replacement and refined to a resolution of 1.9 Å (Table 1). DmTailor^180–560^ adopts a conserved bi-lobed nucleotidyl transferase fold consistent with other previously determined structures of TUTases (36,37,40,42,55–57) (Figure 1C). A DALI structural alignment (48) of DmTailor with *Homo sapiens* TUT7 (PDB ID: 5w0m) and *Schizosaccharomyces pombe* Cid1 (PDB ID: 4nku) yields superpositions with root mean square deviation (rmsd) of 2.6 Å (over 372 residues) and 2.8 Å (over 320 residues), respectively (Supplementary Figure S1). The N-lobe, also known as the catalytic domain, (Figure 1C, colored in blue) is composed of five β-sheets and two α-helices and contains three catalytic aspartate residues Asp278, Asp280 and Asp343. The C-lobe, also termed the central domain, (Figure 1C, colored in yellow) is formed by six α-helices. The UMPNPP substrate is bound together with a single Mg^2+^ ion in the active site located in a cleft at the interface of the N- and C-lobes (Supplementary Figure S2A). Multiple interactions facilitate donor substrate binding and contribute to its specificity (Figure 1D). Tyr390 forms a π-stacking interaction with the uracil base of UMPNPP, while the Mg^2+^ ion is coordinated by oxygen atoms of the α, β and γ phosphates, Asp280, and two water molecules. His522 forms a direct hydrogen bond with O4 of the uracil base. This conserved interaction is a hallmark distinguishing TUTases from PAPs that has been shown in Cid1 to determine specificity for the UTP donor (40,55). Thus, the DmTailor^180–560^–UMPNPP structure reveals a conserved mechanism of donor substrate recognition consistent with its activity selectively adding oligo-U tails to the 3′ ends of RNA substrates (32,33).

**Table 1.** Crystallographic data collection and refinement statistics

Dataset	Tailor – UMPNPP	Tailor – GpU	Tailor – CACAGU	Tailor – U_6_
X-ray source	SLS PXIII	SLS PXIII	SLS PXIII	SLS PXIII
Space group	*P*3_1_21	*P*3_1_21	*P*3_1_21	*P*3_1_21
Cell dimensions				
*a, b, c* (Å)	61.18, 61.18, 167.38	60.42, 60.42, 162.28	60.08, 60.08, 162.29	60.78, 60.78, 167.22
*α, β, γ* (^o^)	90.00, 90.00, 120.00	90.00, 90.00, 120.00	90.00, 90.00, 120.00	90.00, 90.00, 120.00
Wavelength (Å)	1.00767	1.00003	1.00004	1.00000
Resolution (Å)*	44.77–1.90 (2.01–1.90)	44.00–2.00 (2.07–2.00)	43.80–1.85 (1.96–1.85)	44.55–2.00 (2.12–2.00)
*R*_sym_ (%)*	11.9 (155.0)	11.0 (238.2)	8.8 (270.6)	12.8 (290.1)
*CC1/2 (%)*	99.9 (53.4)	100.0 (39.2)	100.0 (42.1)	100.0 (48.4)
*I*/σI*	15.81 (1.40)	18.48 (1.17)	24.17 (1.13)	17.95 (1.11)
Completeness (%)*	100.0 (100.00)	100.0 (100.0)	99.9 (99.7)	100.0 (99.9)
Redundancy*	19.6 (19.0)	16.5 (16.5)	19.3 (18.9)	19.7 (20.6)
**Refinement**				
Resolution (Å)	44.77–1.90	37.61–2.00	43.80–1.85	44.55–2.00
No. reflections	29527	24045	29819	25042
*R*_work_ */R*_free_	0.188/ 0.219	0.204/ 0.233	0.195/ 0.226	0.206/ 0.241
**No. atoms**				
Protein	5631	5687	5660	5718
Nucleic acid	-	63	130	118
Ion/ligand	41	-	2	2
Water	221	91	130	99
***B*-factors**				
Mean	38.0	55.1	46.3	62.1
Protein	37.9	54.4	45.7	61.6
Nucleic acid	-	115.8	65.6	81.4
Ion/ligand	32.8	-	40.4	45.3
Water	40.0	50.5	45.1	62.1
**R.m.s. deviations**				
Bond lengths (Å)	0.009	0.007	0.008	0.004
Bond angles (^o^)	1.06	0.86	0.95	0.64
**Ramachandran plot**				
% favored	98.8	98.3	99.4	98.6
% allowed	1.2	1.7	0.6	1.4
% outliers	0.0	0.0	0.0	0.0
**Molprobity**				
Clashscore	2.3	5.2	4.1	4.6

*Values in parentheses denote highest resolution shell.

In addition to the N- and C-lobes of the catalytic domain, DmTailor^180–560^ also contains a structured N-terminal extension (residues 195–220) that is oriented differently relative to the catalytic domain than the N-termini of TUT7 or Cid1 (Supplementary Figure S1). The extension occupies a position similar to that of the C-terminal region of TUT7, which contains a zinc knuckle domain downstream of its nucleotidyl transferase domain. Zinc finger domains are present in a subset of TUTases where their function does not appear to be conserved. Whereas the zinc finger and zinc knuckle domains of TUT4 or the zinc finger of TUT1 contribute to substrate RNA recognition and hence TUTase activity (14,36), the zinc finger domain in the trypanosomal TUTase RET1 has no effect on catalytic activity but is instead required for the correct folding of the catalytic core (58,59). Although the putative C2H2 zinc finger motif of DmTailor is absent from the construct used for crystallization (Figure 1A), structural superposition of DmTailor^180–560^ and TUT7 (Supplementary Figure S1A through C) suggests that the DmTailor zinc finger domain could be similarly positioned with respect to the nucleotidyl transferase domain. Notably, however, the DmTailor zinc finger domain is not required for uridylation activity *in vitro* and *in vivo* (30,31). It is instead involved in mediating interactions with DmDis3L2 along with the DUF1439 domain (31), although the DUF1439 domain alone is sufficient for the interaction (30). The DmTailor^180–560^ structure hints that the zinc finger domain might be involved in coupling the TUTase and nuclease activities of DmTailor and DmDis3L2 within the TRUMP complex; however, further studies will be required to confirm this hypothesis.

### Structures of tailing product complexes reveal the mechanism of acceptor substrate recognition

**Figure 2.**
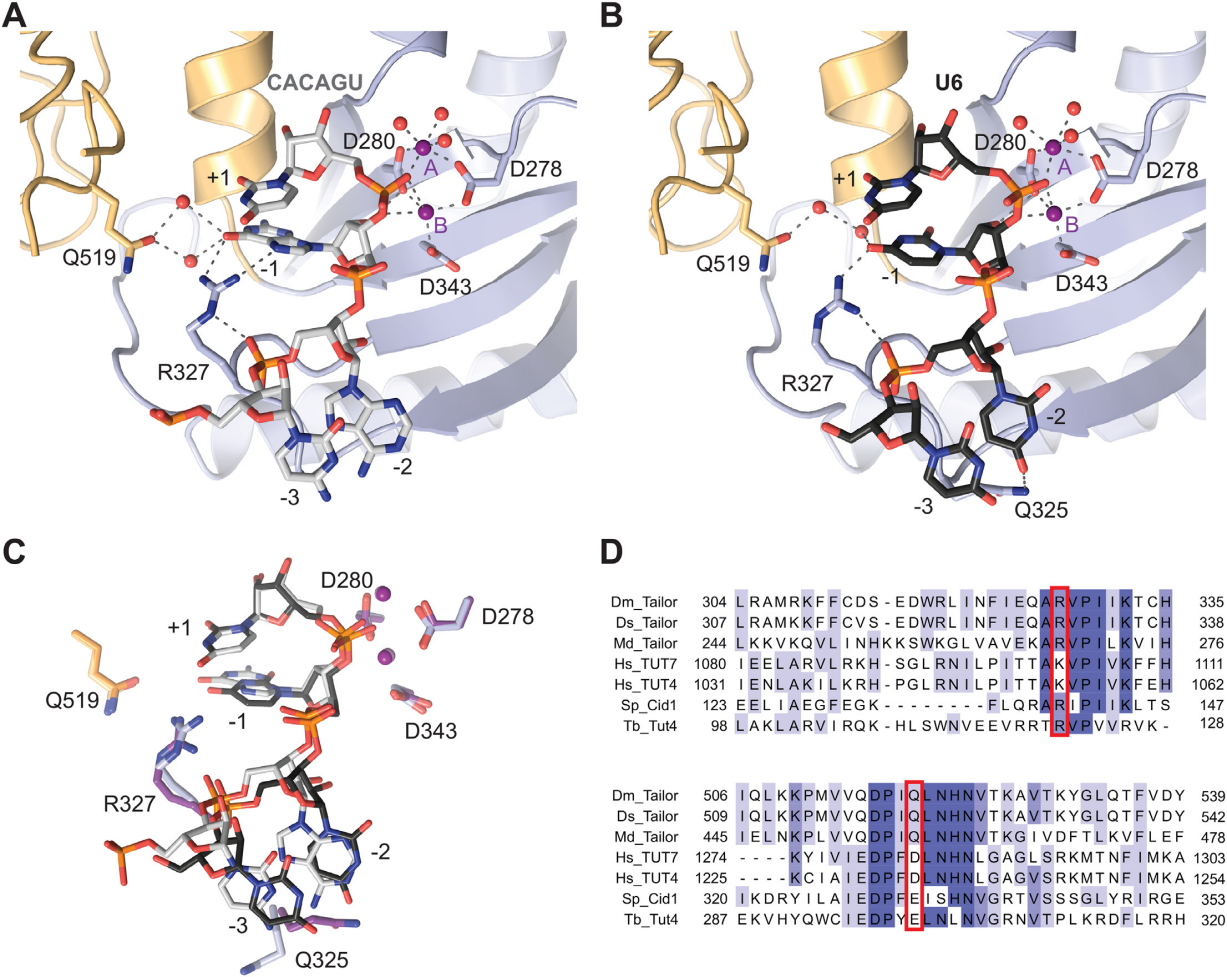
Structures of product-bound DmTailor complexes. (**A**) Zoom-in view of the active site of DmTailor bound to a CACAGU hexanucleotide (grey sticks) and two Mg^2+^ ions (purple spheres). DmTailor lobes are colored as in Figure 1. The catalytic aspartates and the Arg327 and Gln519 residues are shown in stick format, hydrogen bonds and metal coordination bonds are shown as grey dashed lines, water molecules are shown as red spheres. (**B**) Zoom-in view of the active site of DmTailor bound to a U_6_ hexanucleotide (black sticks) and two Mg^2+^ ions (purple spheres). (**C**) Superposition of the CACAGU and U_6_ complex structures in (A) and (B). The structures were superimposed using DALI (48). The structures are colored as in (A) and (B) with the residues and magnesium atoms of the U_6_-bound DmTailor in darker colors. (**D**) Sequence alignment of *Drosophila melanogaster* Tailor (Dm_Tailor*), Drosophila sechellia* Tailor (Ds_Tailor), *Musca domestica* Tailor (Md_Tailor), *Homo sapiens* TUT7 (Hs_TUT7), *Homo sapiens* TUT4 (Hs_TUT4), *Schizosaccharomyces pombe* Cid1 (Sp_Cid1) and *Trypanosoma brucei* Tut4 (Tb_Tut4). The sequences were aligned using MAFFT version 7 (51). Conserved residues are highlighted in darker shades of blue with increasing degree of conservation (invariant in dark blue). Putative residues involved in acceptor substrate specificity, Arg327 and Gln519, are highlighted using red boxes. For a sequence alignment of the full nucleotidyl transferase domain, see Supplementary Figure S4.

DmTailor is the only TUTase so far reported to selectively uridylate RNAs with a 3′-terminal guanosine (32,33). To gain insight into its RNA substrate specificity, we initially co-crystallized DmTailor^180–560^ in complex with the dinucleotide GpU, which acts as a monouridylated product mimic. The structure of the DmTailor^180–560^–GpU complex (DmTailor^180–560^–GpU) was solved at a resolution of 2.0 Å (Table 1, Supplementary Figure S2B and C). As shown by the composite annealed omit map (Supplementary Figure S2B), the electron density for the guanosine moiety was weak possibly due to weak binding. Nevertheless, it was possible to unambiguously build the GpU dinucleotide in the active site. Superposition of the DmTailor^180–560^–UMPNPP and DmTailor^180–560^–GpU structures reveals that the uridine nucleotides at position +1 adopt the same position in the active site. The guanine base at position -1 forms a π-stacking interaction with the uracil base (Supplementary Figure S2C) and the O6 atom is contacted by the guanidine group of Arg327 via hydrogen bonds. No other residue is within hydrogen-bonding distance of the guanosine nucleotide in the DmTailor^180–560^-GpU structure and the only other interaction, between O6 and the Gln519 residue, is mediated via a water molecule. To extend our investigations of acceptor substrate recognition by DmTailor, we subsequently co-crystallized DmTailor^180–560^ with the hexanucleotide CACAGU and determined the structure of the complex at a resolution of 1.85 Å (Figure 2A). The 3′-terminal nucleotides CAGU are clearly defined in electron density maps (Supplementary Figure S2D). Superposition of the UMPNPP- and CACAGU-bound structures reveals similar positioning of the uridine nucleotides at the +1 position in the active site (Supplementary Figure S2E). We therefore conclude that the structure of the DmTailor^180–560^–CACAGU complex represents a product-bound state after monouridylation of a 3′-terminal G RNA substrate. In contrast to the GpU-bound complex, the guanine base at position –1 is contacted by the guanidine group of the Arg327 side chain via a bidentate hydrogen-bonding interaction, due to slight repositioning of the Arg327 side chain (Supplementary Figure S2F). As a result, Arg327 not only provides base-specific recognition of the guanine base but also makes an electrostatic interaction with the phosphate group of the nucleotide at the –2 position, thereby likely contributing to substrate affinity.

Besides the CACAGU RNA, the structure additionally contains two Mg^2+^ ions in the active site. The first Mg^2+^ ion (labeled A in Figure 2A), which corresponds to the Mg^2+^ ion in the UMPNPP-bound complex, is coordinated by the phosphate oxygen of the +1 nucleotide, and by Asp280, Asp278 and three water molecules. The second Mg^2+^ ion (labeled B in Figure 2A) is coordinated by all three of the catalytic aspartates (Asp278, Asp280, Asp343) as well as by the phosphate oxygen of the +1 nucleotide and the 3′-hydroxyl group of the -1 nucleotide. Compared to the UMPNPP complex structure, the side chain of Asp278 adopts a different rotamer conformation in the CACAGU-bound complex due to the coordination of the two Mg^2+^ ions (Supplementary Figure S2E). The presence of two Mg^2+^ ions in the active site is in contrast with previously determined structures of product-bound TUTases (36,38,42), in which no or only one Mg^2+^ ion is present. The position of the second Mg^2+^ ion is, however, consistent with the substrate binding mode observed previously for *Trypanosoma brucei* Tut4 (TbTut4) (42), whereby the interaction between the second Mg^2+^ ion and the 3′-hydroxyl of the incoming RNA is responsible for the correct positioning of the substrate for the nucleophilic attack. These observations are thus consistent with the conclusion that the DmTailor^180–560^–CACAGU structure represents a post-catalytic state before the release of the Mg^2+^ ions and the monouridylated RNA product. The electrostatic surface potential (Supplementary Figure S2G) shows the –2 and –3 nucleotides bound in a positively charged groove that contributes to substrate binding and likely directs the incoming substrate to the active site. Furthermore, the catalytic residue Asp280 forms a hydrogen bond with the 2′-hydroxyl of the ribose of the -1 nucleotide, playing essential role in the recognition of RNA substrates, as opposed to DNA.

DmTailor is not only selective for a 3′-terminal guanosine nucleotide but also for uridine, which facilitates the extension of initially monouridylated substrates to ensure the generation of an oligo-uridine tail required for subsequent degradation by DmDis3L2 (30,33). To obtain insights into mechanism of substrate recognition during oligouridylation, we co-crystallized DmTailor^180–560^ bound to a uridine hexanucleotide RNA (U_6_) and determined the structure of the resulting complex (DmTailor^180–560^-U_6_) at a resolution of 2.0 Å (Table 1, Figure 2B). Electron density for four 3′-terminal nucleotides of the U_6_ RNA substrate and two Mg^2+^ ions was observed, as shown by a composite annealed omit map (Supplementary Figure S2H). The nucleotide at the –1 position in the DmTailor^180–560^–U_6_ structure is coordinated via a single hydrogen bond between O4 of the uracil base to the Arg327 residue (Figure 2B). Interestingly, the uridine at the -2 position is stabilized via a hydrogen bonding interaction of the O4 of the uracil base with Gln325, which adopts a different conformation in the UMPNPP- and CACAGU-bound complexes. Beyond this, the U_6_ and CACAGU RNAs are otherwise bound in a near-identical manner (Figure 2C). Previous studies of SpCid1 have revealed that TUTase enzymes are conformationally dynamic and undergo major conformational rearrangements upon substrate binding (39,40). In the absence of bound donor or acceptor substrates, apo-SpCid1 adopts a closed conformation, whereby the N-lobe rotates by up to 42° toward the C-lobe, closing the active site and hindering substrate access (39). This has been proposed to facilitate product ejection from the active site. The active site cleft of DmTailor has an open conformation in both substrate donor- and product-bound states, suggesting that the enzyme does not undergo a major conformational rearrangement during catalysis. It is nevertheless conceivable that active site closure occurs upon product release, similar to SpCid1 (39,40).

Overall, the structures of the product-bound DmTailor complexes reveal that Arg327 and Gln519 are found in the vicinity of the base of the -1 nucleotide, which corresponds to the 3′-terminal nucleotide of an RNA acceptor substrate in the pre-catalytic state. This points to their involvement in mediating the 3′-terminal substrate recognition, contributing to the observed 3′-terminal nucleotide selectivity of DmTailor. Arg327 is mostly conserved throughout terminal uridylyl transferases, with the exception of human TUT7 and TUT4 enzymes which contain a lysine residue at this position (Figure 2D). In turn, Gln519 is conserved in insect orthologs of DmTailor but is substituted with a glutamate or an aspartate in evolutionarily more distant TUTases (Figure 2D).

### Specific features of the DmTailor active site contribute to acceptor substrate selectivity

**Figure 3.**
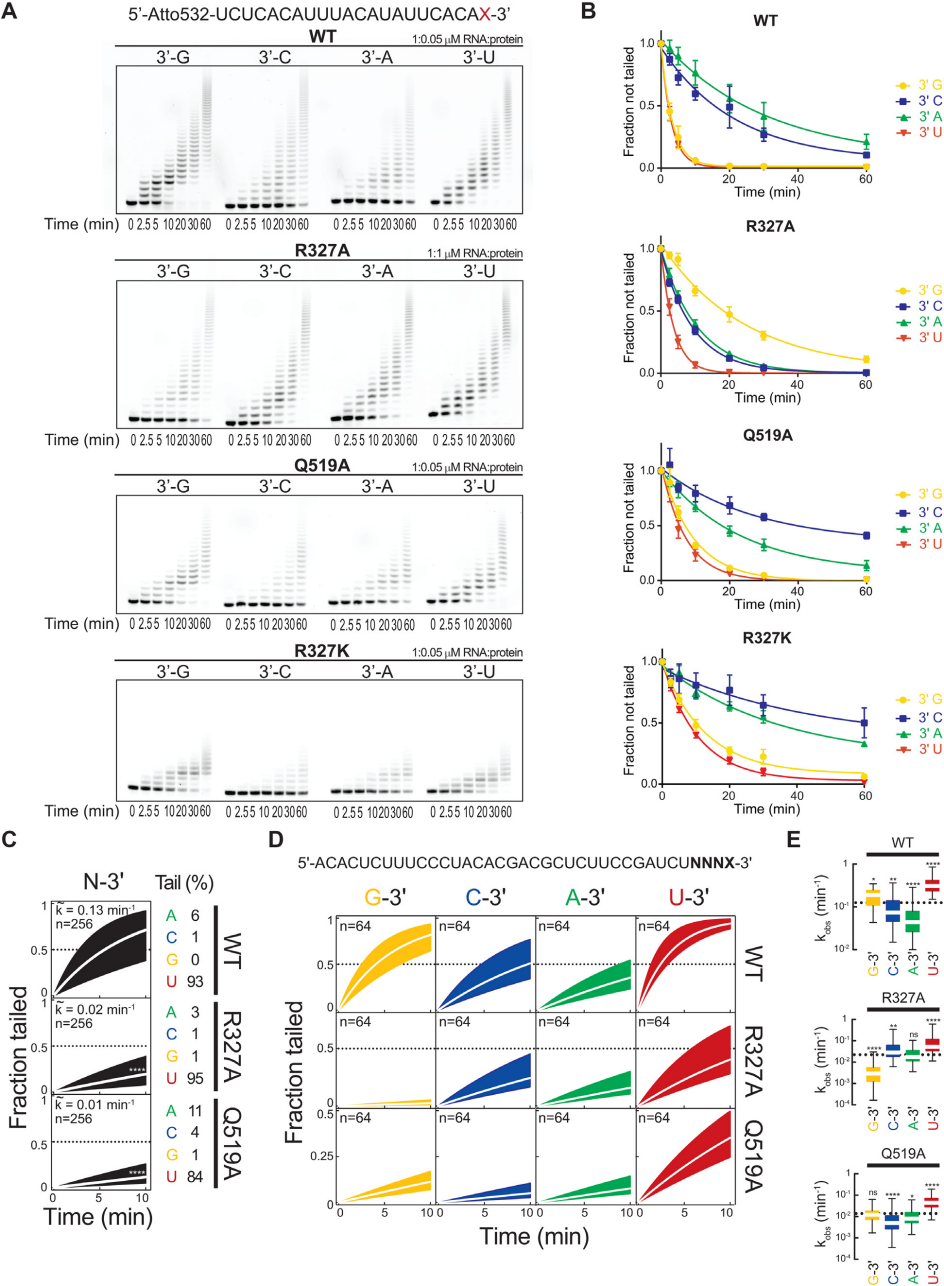
Active site arginine is responsible for substrate specificity of DmTailor. (**A**) *In vitro* uridylation assay with WT DmTailor^180–560^ and the R327A, Q519A, and R327K mutants. Four fluorophore-labeled 22-nt RNA substrates differing only in the terminal nucleotide were used, as indicated. Reaction products were resolved by denaturing polyacrylamide gel electrophoresis and visualized by fluorescence scanning. Representative gels of three replicates are shown. (**B**) Densitometric quantification of three replicates of the uridylation assay in (A). Data were fitted with an exponential one-phase decay equation in GraphPad Prism6. Error bars indicate standard error of the mean (s.e.m.) (C–E) High-throughput sequencing assay of WT DmTailor^180–560^ and the R327A and Q519A mutants. (**C**) Fraction tailed for all substrates (*n* = 256) over time (0, 2, 5 and 10 min) for WT DmTailor^180–560^ (top), R327A (middle) and Q519A (bottom) mutants. The median observed tailing rate (*k*_obs_, white line) and inner quartile range (black area) are shown. The nucleotide composition (%) of the tail at the end of the reaction is indicated. P-Value was determined by Mann–Whitney test relative to WT (****P < 0.001). (**D**) Fraction tailed for all substrates ending in 3′ G, C, A and U (*n* = 64) over time (0, 2, 5 and 10 min) for WT DmTailor^180–560^ (top), the R327A (middle) and Q519A (bottom) mutants. The median (white line) and inner quartile range (colored area) are shown. (**E**) Tukey box plot showing the observed tailing rates (k_obs_) grouped according to terminal nucleotide identity (*n* = 64; outliers omitted). The median k_obs_ for all 256 substrates is represented as a dotted line. *P*-value was determined by Mann–Whitney test relative to all substrates (*P < 0.05, **P < 0.01, ****P < 0.001, ns non-significant).

To test whether Arg327 and Gln519 in DmTailor are involved in recognition of the 3′-terminal nucleotide in substrate RNAs, we generated the R327A, Q519A, and ‘humanized’ R327K DmTailor^180–560^ mutants and investigated their activities in an *in vitro* uridylation assay along with the WT protein. Four 22-nt single-stranded RNA oligonucleotides were used as the reaction substrates; these were based on the sequence of the 3′-terminal arm of the miR-1003 stem–loop and differed only in the identity of the 3′-terminal nucleotide (Figure 3A and B). The RNA-to-protein ratio in the assay was optimized for each protein construct in order to be able to quantify substrate consumption (molar ratio of 1:0.05 for WT, R327K, and Q519A and 1:1 for the R327A mutant). The activities of the mutants were reduced compared to the WT protein, with R327A exhibiting the lowest activity (Figure 3A and B). Comparing the activities on the four different substrates for each protein, WT DmTailor^180–560^ displayed clear selectivity for RNAs with 3′-terminal G or U nucleotides (Figure 3A and B), consistent with previous reports (32,33). Similar specificity was observed for the Q519A mutant, indicating the Gln519 side chain is involved in the uridylation reaction but not in the specific recognition of the 3′-terminal residue. In contrast, the R327A mutant showed clear abrogation of the 3′-G specificity, whereby the 3′-G substrate was uridylated less readily than the 3′-U, 3′-A and 3′-C substrates (Figure 3A and B). Notably, the R327A mutant retained its specificity for the 3′-U substrate. Due to the lack of additional base-specific interactions with the uracil base in the -1 nucleotide binding pocket, we suggest that the 3′-U specificity is retained due to a combination of other factors. In particular, the uracil base cannot be disfavoured by steric exclusion, as compared to purine bases, due to its smaller size. Secondly, it has been postulated that the processivity of DmTailor is dependent on the secondary structure of the substrate, with changes in processivity depending on the length of the uridine tail (32). The +1 nucleotide binding site has high affinity and specificity for the uracil base of the UTP donor substrate. In the absence of UTP, an RNA substrate terminating in a uridine nucleotide can bind in two positions, with the 3′-U either in the –1 (i.e. substrate) or +1 (i.e. product) position, which likely increases the overall affinity for the RNA substrate and contributes to an increased rate of (mono)uridylation. However, further biochemical studies will be necessary to elucidate the exact mechanism of substrate oligouridylation. Interestingly, the R327K DmTailor^180–560^ mutant showed selectivity for 3′-G and 3′-U RNA substrates, similar to the WT enzyme (Figure 3A and B). This suggests that a lysine residue at this position is also able to support 3′-G recognition, by forming hydrogen bonding interactions with the guanine base at the –1 position and/or by contributing to the electrostatic surface potential of the –1 nucleotide binding pocket in a manner that favours 3′-G binding.

To further characterize the WT and mutant Tailor constructs, we subjected each variant to a previously described high-throughput biochemical assay (32). To this end, we employed a pool of 256 distinct 37-nt RNA substrates containing four randomized nucleotides at their 3′ end, which was subjected to *in vitro* tailing in the presence of all four rNTPs at physiological concentrations in order to investigate NTP-selectivity, tailing efficiency, and substrate specificity for each enzyme variant. We incubated the RNA substrate with the recombinant WT, R327A or Q519A DmTailor^180–560^ for 2, 5 and 10 min followed by substrate high-throughput sequencing (HTP-seq). For comparing tailing efficiencies, we performed the *in vitro* assay using the same RNA-to-protein ratio for all constructs (see Materials and Methods for details). WT DmTailor^180–560^ efficiently catalyzed nucleotide addition to the 3′ ends of RNA substrates with marked selectivity for uridine incorporation (93%) (Figure 3C). Alanine substitutions of Arg327 or Gln519 negatively impacted uridylation efficiency (*P* < 10^−4^; Mann–Whitney test) (Figure 3C and Supplementary Figure S3A). Nonetheless, both the R327A and Q519A mutants retained their selectivity for uridine incorporation (95% and 84%, respectively) indicating that they behave as bona-fide uridylyl transferases under the assay conditions (Figure 3C). WT DmTailor^180–560^ exhibited significantly higher tailing efficiency for substrates with a 3′-terminal G (*P* < 10^−3^; Mann–Whitney test) or U (*P* < 10^−4^; Mann–Whitney test) (Figure 3D and E), in agreement with our PAGE-based activity assays (Figure 3A and B) and previous experiments using immunopurified full-length Tailor (32). In contrast, RNAs ending with 3′-G were the least efficiently tailed substrates for the R327A mutant (*P* < 10^−4^; Mann–Whitney test) (Figure 3D and E). The Q519A mutant tailed 3′-G, 3′-A and 3′-C substrates with comparable efficiencies (Figure 3D and E), suggesting that Gln519 plays a minor but nonetheless accountable role in Tailor's 3′-G specificity. In contrast, the enzymatic preference for substrates with a 3′-terminal U relative to substrates with 3′-C or 3′-A was maintained for both R327A and Q519A Tailor mutants (Figure 3D and E), in agreement with PAGE-based TUTase assays (Figures 3A and B).

The HTP-seq approach allowed us to address the RNA substrate selectivity along the four 3′-terminal nucleotides in the substrate RNA. To investigate the effect of the identity of each nucleotide at each position on the activity of Tailor, we grouped all substrates according to their 4-mer sequence and calculated relative tailing rates (*k*_obs_) for each group (Supplementary Figure S3B, top panel). Calculation of the fold-change in *k*_obs_ for mutant relative to WT DmTailor^180–560^ shows that substrates ending in a 3′-G (NNNG-3′) comprise the RNA group that is least efficiently tailed by the R327A mutant (by <4-fold) (Supplementary Figure S3B, bottom panel). In contrast, mutating residue Gln519 had little impact on tailing rates relative to WT DmTailor^180–560^ for any of the substrate groups (Supplementary Figure S3B, bottom panel). These analyses suggest that tailing efficiencies are mostly affected by the identity of the terminal nucleotide of the RNA substrate. We therefore conclude that Arg327 modulates Tailor's uridylating efficiency based on the identity of the 3′-terminal nucleotide of the acceptor RNA substrate, thereby determining Tailor's enzymatic specificity for substrates with a terminal guanosine.

### Structural comparisons suggest 3′-G acceptor substrate selectivity in other TUTases

Taken together, our results show that a single amino acid residue, Arg327, is responsible for the selectivity of DmTailor toward 3′-G RNA substrates but does not affect its 3′-U specificity nor its uridylyl transferase specificity. Although Arg327 is strongly conserved throughout other TUTases (Figure 2D), DmTailor is the only TUTase that has been reported to show 3′-G RNA substrate specificity to date, which allows Tailor to preferentially uridylate mirtron RNAs. Mirtron uridylation is conserved in other Drosophilids (60), along with the conservation of Arg327 in Tailor orthologs in these species (Figure 2D, Supplementary Figure S4).

Superposition of the crystal structure of HsTUT7 bound to U5 RNA (PDB ID: 5w0m) with the DmTailor^180–560^–U_6_ complex shows that Lys1103^TUT7^, corresponding to Arg327 in DmTailor, is not positioned within hydrogen-bonding distance from the uridine nucleotide (Supplementary Figure S5A) and no involvement in the uridylation activity has been proposed for this residue (36). However, as there are no available crystal structures of HsTUT7 or its paralog HsTUT4 bound to RNAs containing a guanosine at the –1 position, it is unclear whether HsTUT4/7 would recognize a 3′-G RNA in a manner similar to that of DmTailor. The cellular substrates of HsTUT4/7 are diverse and include mRNAs (19), microRNAs (61), pre-microRNAs (62) or non-coding RNAs (35) and 3′-terminal nucleotide specificity has not been demonstrated to date. However, a previous study investigating the uridylation of mRNA poly-A tails by both HsTUT7 and HsTUT4 showed increased uridylation of substrates containing 3′-G compared to those with 3′-A (19). As Lys1103^TUT7^ could conceivably mediate equivalent hydrogen-bonding interactions with the guanine base as for Arg327 in DmTailor, it is possible that HsTUT4/7 possess similar 3′-terminal guanosine specificity as DmTailor. This hypothesis is further strengthened by the results of our *in vitro* uridylation assay (Figure 3A and B) showing that the ‘humanized’ R327K DmTailor^180–560^ mutant exhibits similar 3′-G and 3′-U selectivity as the WT enzyme. Since mammalian mirtrons undergo extensive 3′ uridylation (63), the putative 3′-G selectivity of mammalian TUTases such as TUT4/7 could be a contributing factor. Furthermore, mammalian 3′-polyadenylated mRNAs were recently shown to undergo guanylation by TENT4A (PAPD7) and TENT4B (PAPD5) enzymes (64), which has a stabilizing effect due to the refractoriness of 3′-G RNAs to deadenylases such as the CCR4-NOT complex. Selective uridylation of guanine-terminated mRNAs could thus provide a mechanism for the degradation of 3′-G mRNAs by triggering uridylation followed by Dis3L2-mediated decay.

Like HsTUT4/7, the yeast TUTase Cid1 also oligouridylates mRNAs, thus inducing their degradation (17). Superposition of the DmTailor^180–560^-U_6_ structure with ApU-bound SpCid1 (PDB ID: 4nku) reveals that the loop containing Arg139^Cid1^, the residue corresponding to DmTailor Arg327, is displaced in a manner that positions the Arg139^Cid1^ side chain away from the –1 nucleotide binding pocket (Supplementary Figure S5B). This loop displacement suggests a mechanism by which the binding site can accommodate oligoadenylated mRNA substrates. To date, structures of SpCid1 bound to RNA substrates with non-adenosine nucleotides at the -1 position are not available. However, a previous study showed that the R319A^Cid1^ mutant has decreased activity on both UpU and A15 but did not test other RNA substrates (37). Interestingly, the structure of TbTut4 in complex with UTP and UMP (PDB ID: 2q0f), in which the UMP molecule is coordinated at the –1 position, revealed that Arg121^Tut4^, corresponding to Arg327 in DmTailor, hydrogen-bonds to O4 of the UMP base (Supplementary Figure S5C), prompting the conclusion that Arg121^Tut4^ determines the selectivity for a 3′-uridine substrate over a 3′-adenosine (42). The R121A^Tut4^ mutant exhibits a 100-fold decrease in the catalytic rate of uridylation (57), consistent with our biochemical assays of DmTailor TUTase activity (Figure 3A and C). However, the activity of TbTut4 on 3′-G substrates has not been tested thus far.

Therefore, based on our structural observations and biochemical experiments, and in light of previous studies of other TUTases, we propose that the 3′-terminal guanosine substrate selectivity might not be unique to DmTailor. Instead, the near-universal conservation of basic residues (Arg or Lys) in the –1 nucleotide binding pocket points to a conserved mechanism that might confer selectivity for 3′-G RNA substrates to numerous eukaryotic TUTases. Confirmation of this hypothesis awaits further detailed systematic investigations of the substrate preferences within the TUTase enzyme family.

## CONCLUSIONS

In this work, we set out to investigate the molecular basis for the selectivity of Tailor TUTases toward RNAs containing 3′-terminal guanosine or uridine nucleotides. By determining crystal structures of DmTailor in complexes with UTP donor or mono- and oligo-uridylated RNA product mimics, we identified specific features of the enzyme's active site that mediate base-specific interactions both with the UTP donor and RNA acceptor substrates, thereby ensuring selective uridylation of RNA substrates terminating with 3′-G or 3′-U. Conservation of these active site features in other TUTases suggests that 3′-G selectivity might be a general property of many TUTases, including human TUT4/7, thus having functional implications for their roles in regulating the processing and stability of the transcriptome. Together, these studies shed light on the mechanism of Tailor in uridylation-based RNA surveillance and decay in *Drosophila* and advance our general understanding of the molecular mechanisms of TUTases in post-transcriptional gene expression control.

## DATA AVAILABILITY

Atomic coordinates and structure factors for the reported crystal structures have been deposited with the Protein Data Bank under accession numbers 6I0S (DmTailor-UMPNPP), 6I0T (DmTailor-GpU), 6I0U (DmTailor-U6) and 6I0V (DmTailor-CACAGU).

## Supplementary Material

Supplementary Data
