# Supplementary material for: Structural basis for acceptor RNA substrate selectivity of the 3′ terminal uridylyl transferase Tailor
Source: Nucleic Acids Res. 2018 Nov 20;47(2):1030–42. doi: 10.1093/nar/gky1164 (PMC6344859; doi:10.1093/nar/gky1164)
Supplement: Supplementary Data [file gky1164_supplemental_files.pdf]

SUPPLEMENTARY MATERIAL

Supplementary Table S1. RNA oligonucleotides used as substrates for the in vitro tailing assay and the sequencing-based activity assay.

**Name**	**RNA sequence (5'-3')**
In vitro tailing assay (Figures 1B, 3A and B)	
miR-1003-3p-G	/Atto532/UCUCACAUUUACAUAUUCACAG
miR-1003-3p-C	/Atto532/UCUCACAUUUACAUAUUCACAC
miR-1003-3p-A	/Atto532/UCUCACAUUUACAUAUUCACAA
miR-1003-3p-U	/Atto532/UCUCACAUUUACAUAUUCACAU
Sequencing-based activity assay (Figures 3C through E, S3)	
	ACACUCUUUCCCUACACGACGCUCUUCCGAUCUNNNN

Supplementary Table S2. Sequencing statistics for data analysis of the sequencing-based activity assay.

Sample	Time (min)	Reads	Adapter stripped	Unambig. reads (excluding N)	Background corrected	Tailed reads	Tail composition (%)			
							A	C	G	U
Input replica 1	-	1678124	1676875	1355549	1351772	0	n.a.	n.a.	n.a.	n.a.
Input replica 2	-	1786361	1785339	1441297	1436873	0	n.a.	n.a.	n.a.	n.a.
WT replica 1	2	1614557	1612739	1298556	1272487	378653	4.71	1.48	1.05	92.76
WT replica 1	5	1897449	1896097	1524957	1479500	818476	5.06	1.28	0.64	93.02
WT replica 1	10	1582889	1581477	1269160	1213452	870075	5.64	1.05	0.24	93.07
WT replica 2	2	1981411	1980081	1591584	1559193	445931	4.42	1.37	0.96	93.24
WT replica 2	5	1153508	1152601	931075	902811	496220	4.88	1.20	0.56	93.37
WT replica 2	10	1578224	1576770	1267825	1212516	886644	5.62	1.11	0.30	92.97
R327A replica 1	2	1424429	1423255	1149538	1136994	101344	5.11	3.22	3.95	87.72
R327A replica 1	5	1408915	1408113	1123412	1109150	175487	2.75	1.49	1.56	94.20
R327A replica 1	10	1639650	1636827	1319832	1299428	314724	2.38	1.15	1.05	95.42
R327A replica 2	2	1083930	1083105	876592	866978	73464	3.20	1.89	2.39	92.52
R327A replica 2	5	1206536	1205915	972912	959563	156285	2.83	1.54	1.65	93.98
R327A replica 2	10	1397387	1396464	1131414	1111735	280611	2.82	1.45	1.38	94.35
Q519A replica 1	2	997350	996765	794798	787560	5533	18.30	11.23	14.62	55.85
Q519A replica 1	5	1215588	1213569	981097	964621	82989	11.76	4.53	3.07	80.64
Q519A replica 1	10	1152571	1152039	921847	899024	207574	11.30	3.60	1.59	83.51
Q519A replica 2	2	1457476	1456611	1180830	1168650	13538	16.13	8.72	10.40	64.76
Q519A replica 2	5	996508	996107	796426	780248	75964	14.18	6.85	6.37	72.59
Q519A replica 2	10	2319818	2318620	1863680	1812985	425195	11.23	3.52	1.38	83.86

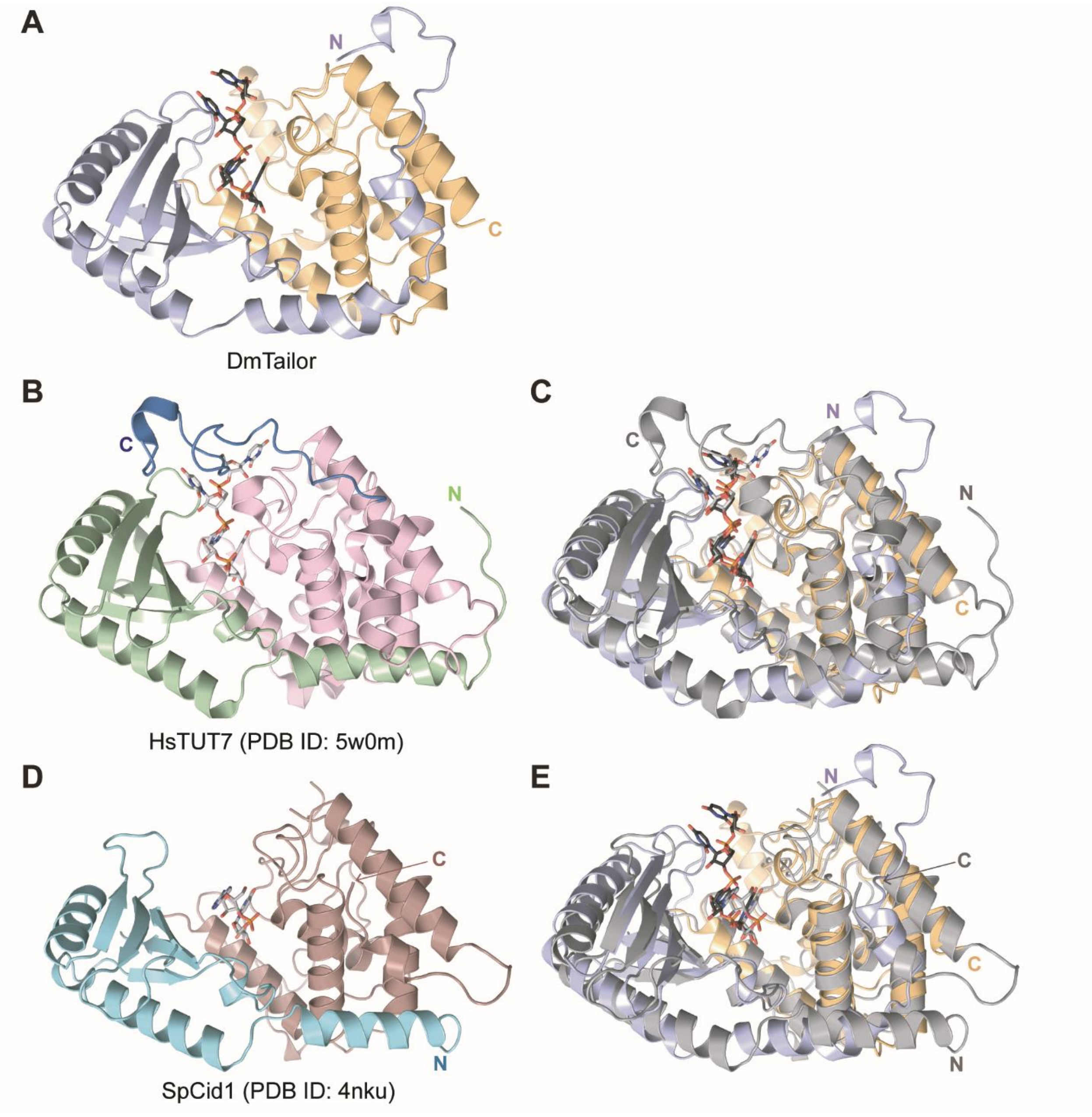

Supplementary Figure S1. Structural superpositions of DmTailor$^{180\text{-}560}$-U6 complex with HsTUT7-U5 (PDB ID: 5w0m) and SpCid1-ApU (PDB ID: 4nku) complexes. **(A)** Cartoon representation of the overall structure of DmTailor bound to U6 (black sticks), colored as in Figure 2. **(B)** Overall fold of HsTUT7-U5 shown in the same orientation as DmTailor-U6 in (A). The N-lobe of HsTUT7 is colored green, the C-lobe pink, the zinc knuckle 2 in dark blue and U5 is shown as grey sticks. **(C)** DALI superposition of DmTailor-U6 and HsTUT7-U5. DmTailor is colored as in (A), HsTUT7 in grey. **(D)** Overall fold of SpCid1-ApU shown in the same orientation as DmTailor-U6 in (A). The N-lobe is colored cyan, the C-lobe brown, the ApU substrate is shown as grey sticks. **(E)** DALI superposition of DmTailor-U6 and SpCid1-ApU. DmTailor is colored as in (A), SpCid1 is shown in grey.

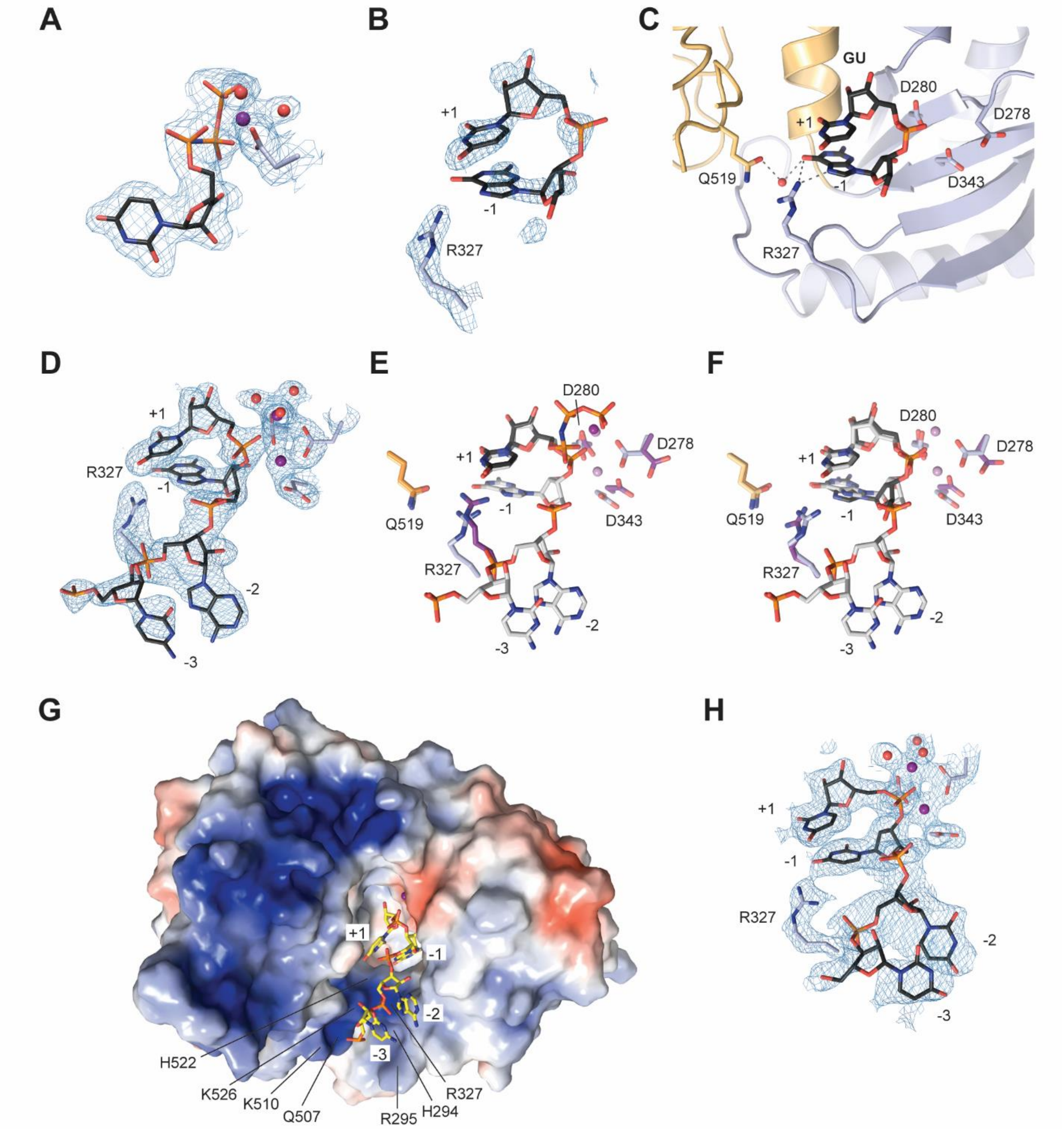

Supplementary Figure S2. (A) Composite annealed omit map of UMPNPP bound to DmTailor$^{180\text{-}560}$, contoured to 1 σ. The omit map electron density for residues involved in metal coordination as well as Arg327 are also shown. Mg^{2+} ions are depicted as purple spheres, water molecules as red spheres. **(B)** Composite annealed omit map of GpU bound to DmTailor$^{180\text{-}560}$, contoured to 1 σ. **(C)** GpU dinucleotide (black sticks) bound in the active site of DmTailor colored as in Figure 1. The catalytic aspartates and the Arg327 and Gln519 residues are shown in stick format, hydrogen bonds are shown as grey dashed lines, water molecules are shown as red spheres. **(D)** Composite annealed omit map of CACAGU bound to DmTailor$^{180\text{-}560}$, contoured to 1 σ. **(E)** Superposition of the UMPNPP- and CACAGU-bound DmTailor structures. UMPNPP is shown in black, CACAGU is shown in grey. The residues and magnesium are colored as in Figure 2 with light and dark colors used for the CACAGU- and UMPNPP- bound DmTailor, respectively. **(F)** Superposition of the GpU- and CACAGU-bound DmTailor$^{180\text{-}560}$ structures. GpU is shown in black, CACAGU is shown in grey. The residues and magnesium are colored as in Figure 2 with light and dark colors used for the CACAGU- and GpU-bound DmTailor, respectively. **(G)** Electrostatic charge distribution of DmTailor$^{180\text{-}560}$-CACAGU. The surface potential was calculated using the Adaptive Poisson-Boltzmann Solver (APBS) plugin (50) in PyMol (Schrödinger, LLC), with positive charge shown in blue, negative in red, neutral in white. The CACAGU RNA is shown as yellow sticks. **(H)** Composite annealed omit map of U6 bound to DmTailor$^{180\text{-}560}$, contoured to 1 σ.

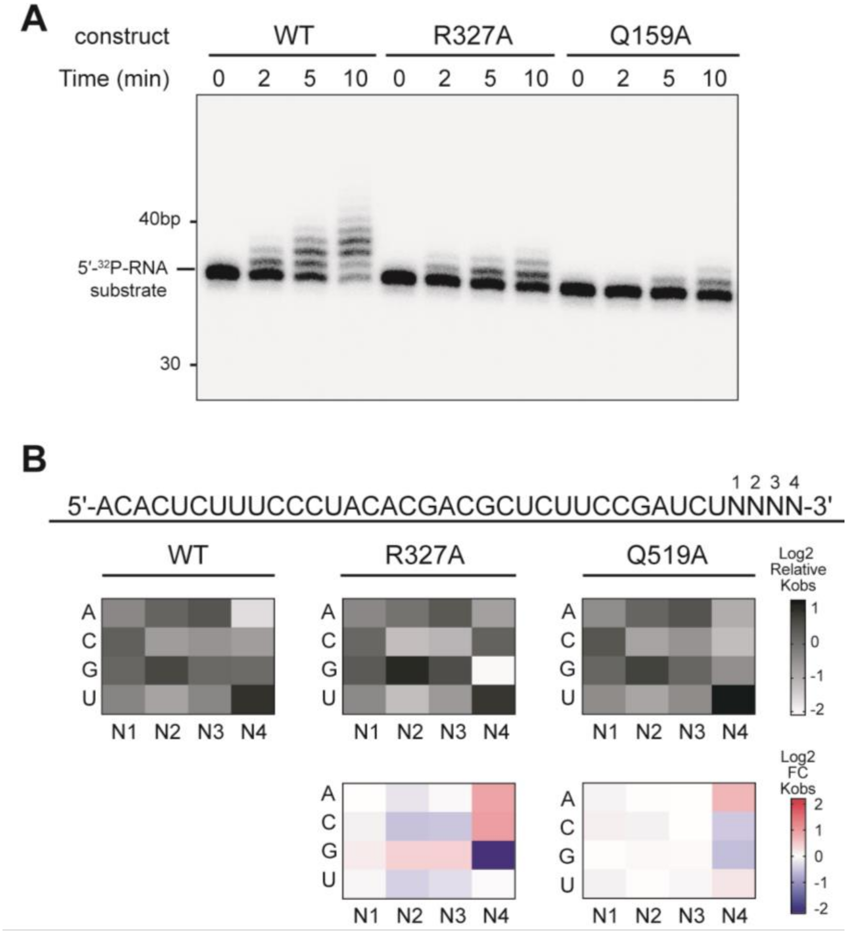

Supplementary Figure S3. High-throughput sequencing assay. **(A)** Denaturing PAGE analysis of in vitro tailing reactions for the high-throughput sequencing assay using recombinant WT and mutant DmTailor$^{180\text{-}560}$ in the presence of all four rNTPs at 2, 5 and 10 min. 10 nM 5' radiolabeled RNA and 50nM protein were used. **(B)** Positional analysis of the nucleotide identity of the randomized 3' end of RNA substrates. Heat map (top panel) of the log2 of the relative observed tailing rate (K_{obs}) for all substrates (n=256) grouped according to the identity and position of each nucleotide (each group n=64) within the randomized 3' end of RNA substrates for WT DmTailor$^{180\text{-}560}$ (top left), R327A (top middle) and Q519A (top right) mutants. Lighter-shaded squares indicate decrease in tailing rate compared to all substrates; darker-shaded squares indicate increase in tailing rate compared to all substrates. Heatmap (bottom panel) representing the log2(fold-change) in tailing rate (K_{obs}) for the R327A (bottom left) and Q519A (bottom right) mutants relative to WT DmTailor$^{180\text{-}560}$. Red squares indicate higher relative tailing rate; blue squares indicate lower relative tailing rate; white squares indicate no change in relative tailing rate.

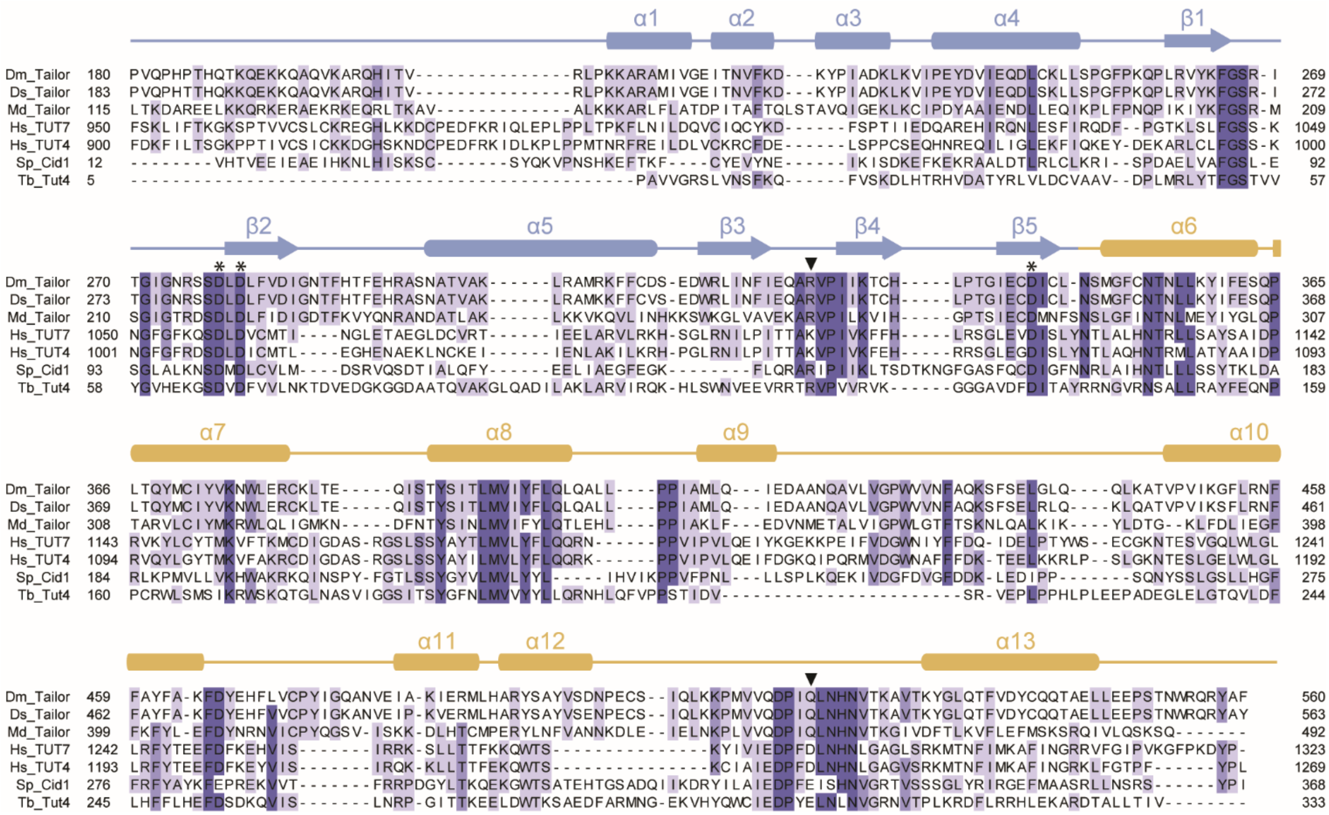

Supplementary Figure S4. Sequence alignment of the nucleotidyl transferase domains of *Drosophila melanogaster* Tailor (Dm_Tailor*), Drosophila sechellia* Tailor (Ds_Tailor), *Musca domestica* Tailor (Md_Tailor), *Homo sapiens* TUT7 (Hs_TUT7), *Homo sapiens* TUT4 (Hs_TUT4), *Schizosaccharomyces pombe* Cid1 (Sp_Cid1) and *Trypanosoma brucei* Tut4 (Tb_Tut4). Conserved residues are highlighted in dark blue. The catalytic triad aspartates (Asp278, Asp280, Asp343) are indicated with a star, residues Arg327 and Gln519 are indicated with a black arrow. The NCBI accession numbers are: AAF54068 (DmTailor), XP_002038641 (DsTailor), XP_019892831 (MdTailor), NP_001171988 (HsTUT7), NP_056084m (HsTUT4), CAB50789 (SpCid1), ABL07000 (TbTut4). Secondary structure features are indicated above the alignment and colored as in Figure 1.

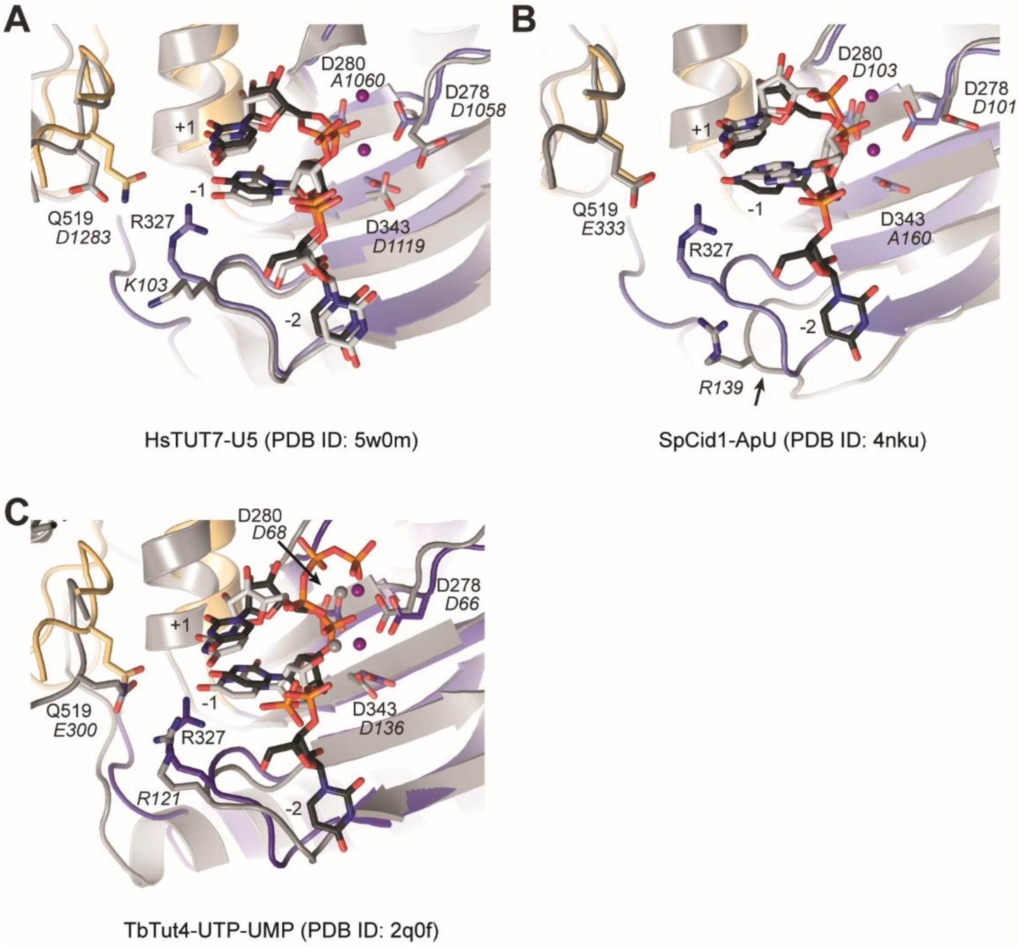

Supplementary Figure S5. Close-up views of the active site of DmTailor$^{180\text{-}560}$-U6 complex superimposed with **(A)** HsTUT7-U5 complex (PDB ID: 5w0m), **(B)** SpCid1-ApU complex (PDB ID: 4nku) and **(C)** TbTut4-UTP-UMP complex (PDB ID: 2q0f). DmTailor is colored as in Figure 2B. The HsTUT7/SpCid1/TbTut4 structures are colored in grey and the residues are labeled in italics. The Arg327-containing loop is highlighted with a black arrow in (B).
